# To Be Remembered

**Published:** 1869-02

**Authors:** 


### TO BE REMEMBERED.

Our readers are especially requested to remember that we often recommend to them, for purchase and use, various articles which will add to their enjoyment and comfort, without any pay, past, present or prospective; and without having any acquaintance whatever, or ever having seen the persons who have those things for sale, with the sole view of making the "Journal" serviceable to its readers. One of these we desire now to speak of is an article for the feet, made out of felt, with sole half an inch thick, and which is perfectly delightful for its warmth in some cases of

COLD FEET,

From which many suffer very much. This felt shoe is to be had at the corner of Broome and Centre streets, New York, in a little tumble-down Dutch shoe shop, and we have never seen them elsewhere. When first noticed, the idea occurred that if worn in the house they would keep the feet too warm, and that putting on the common shoe or boot, on going out of doors, the change would be too great, and a bad cold would be the inevitable result.

But in thinking the matter over, this knock-down argument presented itself: no one can sleep well or feel well when the feet are all the time cold; it renders the whole body uncomfortable, and the mind soon participates; and, if continued, serious and fatal disease will eventually and inevitably attack the system. This being the case, would it not be better for the feet to be warm in the house and cold out of doors, than to be cold all the time; so we determined to try it, as to one of the members of our family, and the result of a whole winter's experiment has been very satisfactory; hence this suggestion; and our readers may rest assured that a great comfort will ensue in many cases to those who will use the old Dutchman's most uncouth and ungainly felt shoe, and perhaps some lady-reader of elegant taste and leisure, or some down-east Yankee may invent a beautiful pattern, having all the advantage without the uncouthness of that of Herr Hans of Center street.

On this same subject of cold feet, a word may be said profitably about

SLIPPERS.

We purchased a pair about six weeks ago of an old woman in front of the Prince Charles Hotel, Heidelberg, Germany, for half of a gold dollar, of which it may be safely said there is not such another sensible pair of slippers in the United States at this present writing.

Our readers will be surprised at the intimation that a Dutchman ever did a sensible thing since the world began, for they are so stupid and so slow; and if a bright idea ever comes into their heads, it is because it has been pounded into them by such repeated and glaring experiences that further resistance to its reception becomes a physical and moral impossibility. The entire slippers were made of cloth, except the soles, and they were lined on the inside with a woolen bed, making them exceedingly grateful to the feet if cold; and preventing that very disagreeable sensation experienced when, coming in from a walk, the shoe or boot is taken off and slippers are put on with cold leather bottoms, sometimes sending a cold shudder through the system, giving rise to stiff joints, to rheumatic attacks, to break-bone fevers and other ailments. If the reader will stop a single moment for reflection, he will very certainly call to mind a more or less frequent occurrence of this same disagreeable sensation, when either there was no opportunity to warm the slippers, or there was supposed to be no time to do it, or that by moving about briskly the feeling of coldness to the soles of the feet would be removed.

A case once came under our own notice where several times changing to cold slippers from warm shoes, persisted in for several weeks, caused such a stiffness in all the joints of locomotion that it was with the utmost difficulty a few steps could be taken, or a turn of the body made in bed.

Another item about the Dutch slippers worthy the attention of the honorable shoe fraternity of this country is this: it is known that a pair of slippers often wear out first at that point behind the big and little toes where the spreading foot bulges out over the edge of the soles; at these points in the cloth slipper, the thoughtful German had sewed on neatly a piece of strong morocco as a protection to the cloth, making these points the last to be worn away, instead of the first. This reminds us of an idea of more effulgent brightness coming from the inventive brain of some doctor in New York.

Passing along Third Avenue some time ago, we saw a woman in a shoe-shop sewing a stocking over a last, which excited our curiosity to the extent of causing us to step in and inquire the motive for so unusual a procedure. Shoes and stockings are always used together, but it is rather a rare occurrence to meet with a stocking in a shoe-store; the lady informed us very good naturedly that a doctor who had no wife, finding that his stockings so soon wore out at the heel and toe, and having had instilled into him from early youth that an ounce of prevention was worth a pound of cure, had conceived of the splendid idea that if pieces of soft buckskin were sewed over the toes and heels of his stockings, they would last indefinitely long, therefore he had brought a new pair to her, with some buckskin, and having explained to her his views, she was endeavoring to carry them out.

### HEALTH AND THRIFT

Oftener go together than most persons are aware of. It is a blessed thing to feel that to-morrow is certainly and abundantly provided for, irrespective of the efforts and profits of to-day; in other words, to be a little forehanded, to have a little spare money always on hand. To attain such a position, it is a necessity for the masses that economy should be practised for quite a number of years; hence, with a little aid of the imagination, the reader may be able to see that there is some connection between

ASH-SIFTERS AND HEALTH,

For in our observation money is a medicine, and making money is both a tonic and a stimulant; it accelerates the circulation; it enlivens the spirits; it lightens the heart; it wakes up a man's energies. Take any man any day who is "hard up"—with whom the world does not go well—who finds he is going backwards pecuniarily, and yet claims are coming upon him which must be met; wants which must be supplied; necessities which cannot be evaded; his rent must be paid; his board bill must be liquidated; the butcher and baker and milkman all are pressing for their respective dues: let a man be ever so hopeful, ever so courageous, ever so brave, in general, there will be moments when the difficulties of his position become so pressing that his manliness almost forsakes him; he starts at his own shadow and well nigh comes to the conclusion that it is useless to struggle longer, and that he "might as well give up." Just at this juncture let a proposition be made, by which with the expenditure of a reasonable amount of bodily activity and mental adroitness, more money can be certainly made than he has ever been accustomed to realize in so short a time, with every guarantee that the dividend will increase with time in an encouraging proportion, why he becomes a new man within the hour; his face wears a new aspect, a new life comes into his eyes; color rushes to the cheeks; the spring of youth is impressed into his steps and there is an elasticity imparted to both mind and body to which he has been long an entire stranger. Therefore money-making in a legitimate way, is a medicine good to take and easy, and to save is the easiest way of money-making; so let us save the refuse of our grates and cooking-stoves and furnaces thus: In Fulton street, New York, near the Old Dutch Church, on the same side, and a few doors nearer Broadway, we have noticed for years an apparatus for separating the unburnt coal from the ashes, out of which a pushing man might have made a large fortune since we first mentioned it in our "Journal" before the war. We have used the same one ever since, and it has certainly saved many a ton of coal. But the lazy, unenterprising owner of the patent had not sense enough to see that the shortest road to a fortune now-a-days is the judicious advertising of a useful article, which every family could employ to advantage. This ash-sifter is made in such a way that you take up a scuttleful from the grate, range or pit, pour it into an opening in the top, and before you can set the empty scuttle down, the ashes are in one box and every particle of unburnt coal not smaller than a pea is deposited in another box; upon this sprinkle some water, remove the white lumps, and the remainder is an admirable fuel for the furnace or for other purposes; we think that a family burning fifteen or twenty tons in a year would thus save an amount of coal equal to two or three, and the apparatus can be sold at a good profit for two or three dollars.

